# Real time spatial cluster detection using interpoint distances among precise patient locations

**DOI:** 10.1186/1472-6947-5-19

**Published:** 2005-06-21

**Authors:** Karen L Olson, Marco Bonetti, Marcello Pagano, Kenneth D Mandl

**Affiliations:** 1 Children's Hospital Informatics Program, Children's Hospital Boston, Boston, Massachusetts, USA; 2 Department of Pediatrics, Harvard Medical School, Boston, Massachusetts, USA; 3 Department of Biostatistics, Harvard School of Public Health, Boston, Massachusetts, USA; 4 Istituto di Metodi Quantitativi, Università Bocconi, Milano, Italy

## Abstract

**Background:**

Public health departments in the United States are beginning to gain timely access to health data, often as soon as one day after a visit to a health care facility. Consequently, new approaches to outbreak surveillance are being developed. When cases cluster geographically, an analysis of their spatial distribution can facilitate outbreak detection. Our method focuses on detecting perturbations in the distribution of pair-wise distances among all patients in a geographical region. Barring outbreaks, this distribution can be quite stable over time. We sought to exemplify the method by measuring its cluster detection performance, and to determine factors affecting sensitivity to spatial clustering among patients presenting to hospital emergency departments with respiratory syndromes.

**Methods:**

The approach was to (1) define a baseline spatial distribution of home addresses for a population of patients visiting an emergency department with respiratory syndromes using historical data; (2) develop a controlled feature set simulation by inserting simulated outbreak data with varied parameters into authentic background noise, thereby creating semisynthetic data; (3) compare the observed with the expected spatial distribution; (4) establish the relative value of different alarm strategies so as to maximize sensitivity for the detection of clustering; and (5) measure factors which have an impact on sensitivity.

**Results:**

Overall sensitivity to detect spatial clustering was 62%. This contrasts with an overall alarm rate of less than 5% for the same number of extra visits when the extra visits were not characterized by geographic clustering. Clusters that produced the least number of alarms were those that were small in size (10 extra visits in a week, where visits per week ranged from 120 to 472), diffusely distributed over an area with a 3 km radius, and located close to the hospital (5 km) in a region most densely populated with patients to this hospital. Near perfect alarm rates were found for clusters that varied on the opposite extremes of these parameters (40 extra visits, within a 250 meter radius, 50 km from the hospital).

**Conclusion:**

Measuring perturbations in the interpoint distance distribution is a sensitive method for detecting spatial clustering. When cases are clustered geographically, there is clearly power to detect clustering when the spatial distribution is represented by the M statistic, even when clusters are small in size. By varying independent parameters of simulated outbreaks, we have demonstrated empirically the limits of detection of different types of outbreaks.

## Background

Public health departments in the United States are beginning to gain timely access to health data, often as soon as one day after a visit to a health care facility [1-3]. Consequently, new approaches to surveillance for disease outbreaks are being developed. These methods require models for baseline patterns and thresholds to detect unusual events [1]. Baseline patterns can be modeled in terms of temporal characteristics, spatial characteristics, or both. When cases are clustered geographically, such as those in the Amoy Gardens apartment complex during the 2003 SARS epidemic [4], an analysis of their spatial distribution may greatly facilitate the detection of a disease outbreak. Methods for both temporal and spatial surveillance have been recently reviewed [5,6].

**Figure 1:**
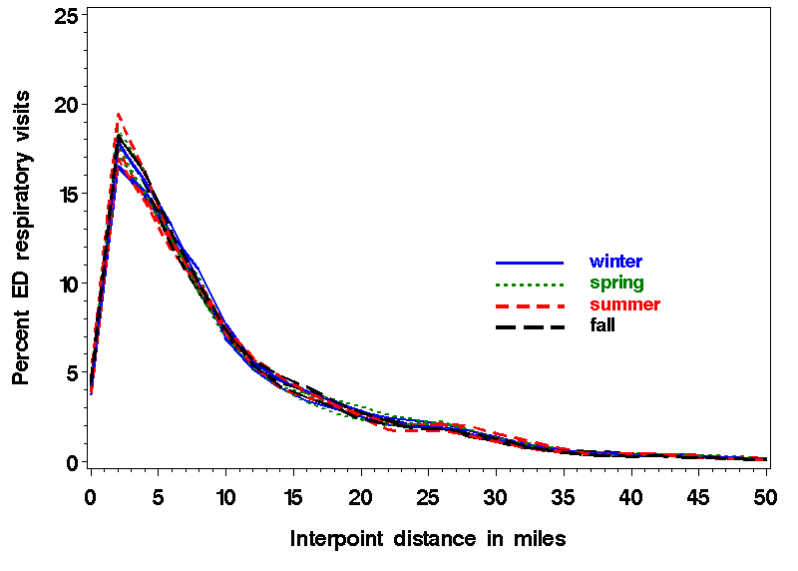
**Pair-wise distances between home addresses of respiratory patients to one hospital over three years by season**. The twelve curves (4 seasons × 3 years) overlap considerably, suggesting stability for the distance distribution over time. The maximum interpoint distance was 100 miles; the distribution up to 50 is shown.

One consideration regarding appropriate baseline data for spatial surveillance is whether to use individual point locations or aggregate counts by regions such as census tracts. Because aggregating points may result in a loss of precision [7], our work uses precise locations, i.e. geocoded patient addresses expressed as longitude and latitude. The novel approach of our method focuses on the detection of perturbations in the distribution of mutual distances among all the individual points in a geographical region to identify clusters [8-10]. Barring outbreaks, this distribution of interpoint distances can be quite stable over time (see Figure 1) [9]. We sought to measure the cluster detection performance of our method, and to determine factors affecting sensitivity to spatial clustering among patients presenting to hospital emergency departments (ED) with respiratory syndromes.

## Methods

This study identifies factors affecting the performance of an algorithm for measuring the degree of deviation from an expected geographic distribution of patient home addresses for a population visiting a localized site of care. The home address is only one of many possible places where a person might be exposed during an actual outbreak. However, other locations are not routinely recorded in the administrative databases typically used for syndromic surveillance. The approach was to (1) define a baseline spatial distribution of home addresses for patients visiting an emergency department with respiratory syndromes using historical data; (2) develop a controlled feature set simulation by inserting simulated outbreak data with varied parameters into authentic background noise, thereby creating semisynthetic data [11]; (3) compare the observed with the expected spatial distribution; (4) establish the relative value of different alarm strategies so as to maximize sensitivity for the detection of clustering; and (5) measure factors which have an impact on sensitivity.

### Study population

Data were obtained retrospectively from hospital databases. The study was approved by the Institutional Review Board. Subjects were ED patients with respiratory syndromes treated at an urban, academic, pediatric, tertiary care hospital from December 24, 2000 to December 20, 2003. These dates were chosen to span the four seasons over three years while maintaining complete seven-day weeks. Patients with respiratory syndromes were identified by chief complaints and diagnostic codes as described in previous reports [1,12]. Of the total of 155,705 ED visits, 28% (43,156) were classified as having respiratory syndromes.

Home addresses of patients were translated to geographic coordinates using ArcGIS 8.1 (Environmental Systems Research Institute, Inc., Redlands, CA). Addresses were cleaned prior to geocoding using software (ZP4, Semaphore Corp., Aptos, CA) that matched addresses to the August 2003 United States Postal Service ZIP+4 database and made corrections. 93% (40,221) of the home addresses were successfully geocoded and patients who lived within 80 kilometers of the hospital (98%) were included in the study, for a total of 39,229 respiratory visits.

**Figure 2:**
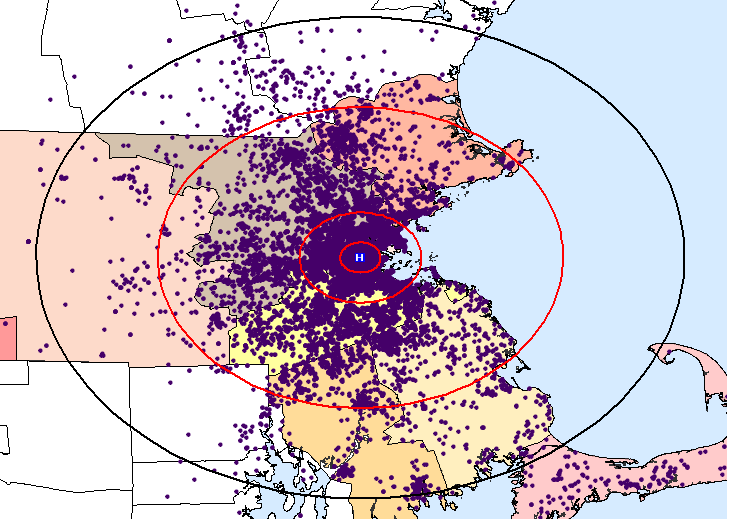
**Baseline distribution of respiratory patients to the emergency department of one hospital**. The study population (blue dots) lived within 80 km of the hospital (black ring). Simulated clusters were placed at 5, 15, and 50 km, along the red rings. Total population density of study patients within the four areas pictured was: 182.6 per square km within 0–5 km of the hospital, 32.6 per sq km within 5–15 km, 1.3 per sq km within 15–50 km, and 0.1 per sq km within 50–80 km.

The number of visits for respiratory syndrome varied by season: 13,156 (34%) in the winter, 9,140 (23%) in the spring, 6,382 (16%) in the summer, and 10,551 (27%) in the fall. The home addresses of study patients were not evenly distributed within the study area. 14,231 (36%) lived from 0–5 km of the hospital, 16,351 (42%) from 5–15 km, 7,545 (19%) from 15–50 km, and 1,102 (3%) from 50–80 km (see Figure 2).

### Baseline spatial distribution of home addresses

The baseline spatial distribution was represented by a set of bins, each containing an equal proportion of pair-wise distance values. To establish this baseline, the three years of data were divided into 156 individual, one-week-long data sets. The number of respiratory visits each week ranged from 120 to 472. The average number varied by season: winter 346 (s.d. 68), spring 229 (s.d. 43), summer 164 (s.d. 28), and fall 271 (s.d. 49). For each week of data, all n(n-1)/2 pair-wise distances among patient addresses were calculated as follows:

*d *= 6378 × 2 × arcsin (), *a *= sin^2 ^((*Y*_1 _- *Y*_2 _)/2) + cos (*Y*_2 _) × cos (*Y*_1 _) × sin^2 ^((*X*_1 _- *X*_2 _)/2),

where Y_1 _and Y_2 _are latitude in radians for point 1 and point 2 of the pair, X_1 _and X_2 _are longitude in radians, and *d *is the interpoint distance in kilometers.

The sets of weekly pair-wise distances were combined into separate data sets by season, and then into a single data set with all seasons combined. Distance values in each data set were ranked in order of magnitude, and divided into ten bins, each with the same number of distance values. To maintain equal proportions, the widths varied (necessarily) across bins. Bin ranges were relatively small for the initial bins (2.6 km on average for bins 1–6), then increased somewhat (9.3 km on average for bins 7–9), with one large final bin (117 km). Next, each distance value in the individual week-long data sets was assigned the bin number into which it fell. For example, if one of the distance values between two patients during a particular winter week was 5 km, then that value fell into bin 3 because its endpoints were from 4.8 to 6.9 km. The number of records in each bin each week was then counted. This resulted in some variability in terms of how many pairs fell into each bin each week, although averaged over all of the weeks, each bin contained 10% of all distance values.

### Controlled feature set simulation

Outbreaks were simulated by adding additional visits to the baseline data. 288 simulated spatial clusters were created using a cluster creation software tool [13]. Each cluster was added to each of the 156 week-long data sets, resulting in 44,928 weekly data sets containing simulated clusters. The clusters varied in size (10, 25, 40 additional visits), distance from the hospital (5, 15, 50 km), and radius of the circle within which points were randomly scattered (0.25, 0.5, 1, 3 km).

In addition, the special situation of an outbreak characterized by an increased number of visits originating over the whole geographic area of interest and, in effect, having no geographic clustering, was studied. Three data sets that varied on size (10, 25, 40 additional visits) were created to simulate this situation. Coordinates were randomly selected from the entire three years of data so that the extra visits would reflect the underlying geographic distribution of the study population. Each data set was inserted into each of the 156 week-long data sets, resulting in 468 weekly data sets containing extra visits dispersed randomly over the entire geographic area.

### Comparison of observed versus expected spatial patterns

A metric, the M statistic, was used to characterize a discrepancy between an expected proportion of distance values in each bin and the actual proportions [9,10] using a nonparametric comparison based on the covariance matrix. Bins endpoints had been defined so that the expected proportions were equal for all bins. The statistic is intended to be sensitive to deviations in the geographic distribution. The M statistic was calculated as follows:

*M *= (*obs *- exp)^*T *^*S*^- ^(*obs *- exp)

where *obs *is a vector of normalized observed proportions, *exp *is a vector of normalized expected proportions, S is a 10 × 10 variance-covariance matrix of the baseline proportions (calculated with data for 156 weeks). T refers to the transpose of the matrix, and S^- ^refers to the Moore-Penrose generalized inverse of the S matrix. Proportions were normalized by dividing the bin frequency by the total for all bins and multiplying by 100.

### Cutoff values

To evaluate the M statistic, cutoff values to indicate clustering were established for each season and for all seasons combined. A simulated baseline data set without extra visits or clusters was used to determine cutoff values at which a false positive alarm rate of .05 could be maintained. This baseline was generated from repeated random samples of patient locations from the week-long data sets described above.

Because the number of ED visits each week varied, sample sizes were generated from a list of weekly visit frequencies. For each season, 1000 frequency values were randomly selected from the weeks that comprised the season. For each of these values, that many addresses were randomly selected from the entire set of actual patient addresses for the season. The M statistic for each data set was calculated, and the 1000 values of the statistic were ranked by magnitude. In separate steps, the 1000 sample sizes were ranked, and the values of M times the sample size were also ranked. This process was repeated for each season. Finally, all seasons were combined and the entire process was repeated, using 5000 instead of 1000 samples for the all-season data. All cutoff values were based on percentile ranks.

### Alarm strategies for the detection of clustering

Six alarm strategies utilizing the M statistic and the number (N) of ED respiratory visits were evaluated. These strategies are listed in Table 1. Each was designed to maintain a false positive rate of 0.05. Two strategies focused only on the number of visits and were included as a comparison to strategies that incorporated spatial information. Because N was not the focus of this study, more complex models for the time series data [14,15] were not investigated. Two strategies evaluated the geographic distribution of patient addresses, and two combined information regarding both the number of visits and the geographic distribution. Four of the six strategies ignored season, and cutoff values were established using the 5000 all-seasons samples. Two strategies required separate values for each season, and the 1000 samples for each season were used to establish these cutoffs. Each alarm strategy was applied to each semisynthetic data set. Sensitivity to the true positive alarms in the data sets with simulated clusters was expected to be high. On the other hand, false alarm rates for data with random additional visits were expected to be near 5% for strategies that evaluated the geographic distribution of visits.

**Table 1:** Description of alarm strategies for the detection of spatial clustering.

Alarm strategy	Description
N > 95^th ^percentile	Number of ED respiratory visits is too high
N > 95^th ^percentile, by season	Number of visits is too high, separate values by season
M > 95^th ^percentile	M statistic is too high
M > 95^th ^percentile, by season	M statistic is too high, separate values for each season
MN > 95^th ^percentile	Calculate M × N, value is too high
N and MN rules	N is too high (top 0.5% distribution) Or M × N is too high (top 0.5% distribution) Or both N is high (>80%) and M × N is high (>80%)

N = number of hospital emergency department respiratory visits, M = M statistic, used to characterize the geographic distribution.

## Results

### Overall performance of the alarm strategies

Overall sensitivity to detect clustering with the addition of simulated geographic clusters is listed in Table 2 by alarm strategy and time of year. Note that use of a single MN cutoff value at the 95^th ^percentile yielded the highest overall sensitivity (62%), and the highest values by season, except for winter, where it was the second best strategy. Therefore, the presentation of results will highlight this strategy, although sensitivity for the other strategies is included in subsequent tables.

**Table 2:** Overall sensitivity to detect spatial clustering.

	Percent of simulated outbreaks that exceeded a threshold				
Alarm strategy	All seasons ^a^	Winter ^b^	Spring ^b^	Summer ^b^	Fall ^b^

N > 95^th ^percentile	8.76	33.33	0.00	0.00	2.56
N > 95^th ^percentile, by season	16.24	11.40	21.67	19.66	11.97
M > 95^th ^percentile	49.17	26.68	53.34	73.71	42.26
M > 95^th ^percentile, by season	49.13	43.61	49.42	55.35	48.01
MN > 95^th ^percentile	62.32	55.43	63.49	70.90	59.27
N and MN rules	55.83	66.60	49.61	55.01	52.52

a The standard errors for All seasons were all less than or equal to 0.2%.

b The standard errors for Winter, Spring, Summer, and Fall were all less than or equal to 0.5%.

Evident in Table 2 is the observation that reliance only on the detection of an increased number of visits was a poor strategy when clusters were relatively small in size. Sensitivity was generally improved by instead relying on the geographic distribution of the clustered points. Sensitivity was most improved when both the increased number and the spatial distribution was incorporated into the alarm strategy.

### Alarm rates when extra visits are not characterized by spatial clustering

A special situation examined in this study was an increase in the number of visits at the same three sizes as the cluster sizes. However, these additional visits were not deliberately characterized by spatial clustering. This scenario could represent either a random increase in visits or an outbreak spread over the entire region. The methods used in this study were not designed to be sensitive to situations where outbreaks do not cluster spatially. Therefore, alarm rates under these conditions could represent either a false alarm rate or a low sensitivity to widely dispersed outbreaks. Rates for each strategy by season are presented in Table 3. The strategies that consider the spatial distribution generally maintained false alarm rates near the desired rate of 5%, with a notable exception of one strategy in the winter.

**Table 3:** Alarm rates for extra visits that are not characterized by geographic clustering.

		Percent of simulated outbreaks that exceeded a threshold				
Alarm strategy	# extra visits per week	All seasons	Winter	Spring	Summer	Fall

N > 95^th ^percentile	10	6.41	23.68	0.00	0.00	2.56
Overall rate = 8.76	25	8.97	34.21	0.00	0.00	2.56
	40	10.90	42.11	0.00	0.00	2.56
						
N > 95^th ^percentile, by season	10	9.62	7.89	12.50	10.26	7.69
Overall rate = 16.24	25	14.74	10.53	20.00	15.38	12.82
	40	24.36	15.79	32.50	33.33	15.38
						
M > 95^th ^percentile	10	3.21	0.00	0.00	12.82	0.00
Overall rate = 2.14	25	3.21	0.00	2.50	10.26	0.00
	40	0.00	0.00	0.00	0.00	0.00
						
M > 95^th ^percentile, by season	10	0.00	0.00	0.00	0.00	0.00
Overall rate = 0	25	0.00	0.00	0.00	0.00	0.00
	40	0.00	0.00	0.00	0.00	0.00
						
MN > 95^th ^percentile	10	4.49	2.63	2.50	12.82	0.00
Overall rate = 3.42	25	5.13	5.26	5.00	5.13	5.13
	40	0.64	2.63	0.00	0.00	0.00
						
N and MN rules	10	7.69	26.32	2.50	0.00	2.56
Overall rate = 7.48	25	7.05	21.05	2.50	0.00	5.13
	40	7.69	23.68	5.00	0.00	2.56

Overall rate is percent positive alarms, regardless of number of extra visits and season. Strategies that only considered N were expected to yield alarm rates greater than the false positive rate of 5% because extra visits were added. Strategies that considered the spatial distribution were expected to yield alarm rates near 5% because the extra visits were not spatially clustered.

### Factors affecting sensitivity to detect clustering

The simulated clusters varied on several parameters. Sensitivity for the four alarm strategies that use the M statistic by cluster size, by distance from the hospital, and by density of the cluster are reported in Tables 4, 5, 6. The two strategies that use only N are not included in these tables because the geographic parameters are ignored by those strategies and results would be identical to those presented in Table 3.

**Table 4:** Sensitivity to detect clustering with simulated clusters of three sizes.

		Percent of simulated outbreaks that exceeded a threshold				
Alarm strategy	# extra visits	All seasons	Winter	Spring	Summer	Fall

M > 95^th ^percentile	10	15.40	1.29	13.28	41.56	5.18
	25	53.75	21.33	62.29	84.94	45.41
	40	78.35	57.43	84.45	94.63	76.20
						
M > 95^th ^percentile, by season	10	8.30	6.30	7.81	11.73	7.32
	25	57.41	47.09	60.34	65.52	56.36
	40	81.69	77.44	80.10	88.81	80.34
						
MN > 95^th ^percentile	10	20.93	13.87	21.43	32.69	15.52
	25	74.69	65.35	76.61	84.70	71.82
	40	91.35	87.06	92.42	95.33	90.46
						
N and MN rules	10	14.86	35.22	6.43	9.56	8.97
	25	65.12	74.67	57.68	67.17	61.38
	40	87.50	89.91	84.71	88.30	87.21

**Table 5:** Sensitivity to detect clustering with simulated clusters at three distances from the hospital.

		Percent of simulated outbreaks that exceeded a threshold				
Alarm strategy	km from hospital	All seasons	Winter	Spring	Summer	Fall

M > 95^th ^percentile	5	34.74	12.76	37.29	63.40	24.89
	15	56.78	33.43	61.73	79.24	51.98
	50	62.17	39.82	67.89	82.91	57.36
						
M > 95^th ^percentile, by season	5	33.20	27.68	30.19	43.76	31.11
	15	57.65	55.64	56.76	65.35	52.84
	50	63.38	54.35	69.55	61.90	67.31
						
MN > 95^th ^percentile	5	49.33	41.82	50.58	60.26	44.42
	15	69.81	62.78	71.01	76.89	68.35
	50	73.41	67.51	74.40	80.13	71.40
						
N and MN rules	5	42.44	56.21	34.10	40.92	39.08
	15	62.87	72.02	57.47	62.91	59.43
	50	67.92	76.03	63.90	67.25	64.80

Patient population density was greatest at 5 km from the hospital and declined as distance away from the hospital increased.

**Table 6:** Sensitivity to detect clustering with simulated clusters of four radius sizes.

		Percent of simulated outbreaks that exceeded a threshold				
Alarm strategy	Radius	All seasons	Winter	Spring	Summer	Fall

M > 95^th ^percentile	250 m	53.94	30.15	59.06	78.45	47.33
	500 m	53.22	29.86	58.23	77.53	46.55
	1 km	51.30	28.07	56.04	75.57	44.80
	3 km	38.22	18.64	40.03	63.28	30.38
						
M > 95^th ^percentile, by season	250 m	54.75	48.10	55.56	61.47	53.67
	500 m	53.82	47.48	54.24	60.58	52.81
	1 km	51.73	45.72	51.81	59.05	50.18
	3 km	36.24	33.15	36.08	40.31	35.36
						
MN > 95^th ^percentile	250 m	66.97	59.54	68.82	75.04	64.25
	500 m	66.41	59.10	67.67	74.96	63.68
	1 km	64.71	57.75	65.94	73.11	61.82
	3 km	51.21	45.32	51.53	60.51	47.33
						
N and MN rules	250 m	60.67	69.52	55.00	61.18	57.34
	500 m	59.93	69.12	54.10	60.33	56.55
	1 km	58.28	67.91	52.47	58.33	54.81
	3 km	44.44	59.87	36.88	40.21	41.38

As shown in Table 4, clusters that are small in size produced the fewest alarms, with an overall sensitivity at size 10 of 21%. However, there was seasonal variability, from a low of 14% in the winter to a high of 33% in the summer. During the winter, when there were the most baseline visits, a cluster of size 10 was about one-seventh the size of the standard deviation for number of weekly visits. In contrast, during the summer, this cluster size was about one-third the standard deviation. With 25 points in the cluster, sensitivity improved markedly to an overall rate of 75%, and 40 clustered points yielded an overall sensitivity of 91%. Again, seasonal variability was evident with the lowest values in the winter and highest in the summer.

In Table 5, the effect of cluster location is demonstrated with clusters placed at three distances from the hospital. Those closest were in regions most densely populated by hospital patients, and were characterized by an overall sensitivity of 49% at 5 km. At greater distances, where the patient population density declined, sensitivity increased to 70% and 73% at 15 and 50 km. Seasonal variability was evident, with winter rates lowest and summer highest.

In Table 6, the effect of cluster dispersion is demonstrated with four radius sizes within which extra visits were randomly scattered. Although sensitivity declined with increasing radius size, the effect was not dramatic at 250 m, 500 m, and 1 km where overall alarm rates decreased from 67% to 65%. However, when the radius increased to 3 km, the decline in sensitivity was greater (51%). Once again, winter rates were lower than summer, and the 3 smallest radii had very similar rates by season.

### Interactions among cluster parameters

To investigate the effects of interactions among the cluster parameters, a logistic regression analysis was performed. Cluster size, distance to hospital, radius size, and all higher order interactions were included in a model to predict whether or not the value of MN exceeded a threshold. All terms were significant, and the maximum-rescaled R-squared was .59. When season and all its interactions with the other variables were added to the model described above, an additional 3-way interaction was significant (cluster size × distance to hospital × season). When this interaction, season, and all two-way interactions with season were added to the first model, the maximum-rescaled R-squared was .61.

To further investigate these interactions, analyses that cross tabulated cluster size, distance from the hospital, and cluster density, and those that cross tabulated these variables with season were performed. Sensitivity values were ranked from highest to lowest to determine which type of cluster produced the least and the most alarms. Overall results are presented in Table 7, and results by season in Table 8.

**Table 7:** Sensitivity to detect clustering by number of extra visits, distance from the hospital, and radius of the simulated cluster.

		Distance from the hospital		
		
# extra visits	Cluster radius	5 km	15 km	50 km
10	250 m	15.58	31.04	25.27
	500 m	14.55	30.59	25.37
	1 km	12.76	30.31	26.28
	3 km	5.90	23.90	24.54
				
25	250 m	68.40	87.27	95.24
	500 m	66.09	87.91	95.33
	1 km	58.27	86.81	95.51
	3 km	26.60	68.86	94.05
				
40	250 m	91.54	99.45	99.82
	500 m	90.45	99.63	99.82
	1 km	88.01	99.63	99.82
	3 km	53.78	92.31	99.82

The numbers in the cells are the percentage of simulated outbreaks that exceeded the 95^th ^percentile value of M × N (M statistic × number of visits).

**Table 8:** Sensitivity to detect clustering by season, number of extra visits, distance from the hospital, and radius of the simulated cluster.

		Distance from the hospital		
		
	Cluster radius	5 km	15 km	50 km
WINTER				

10 extra visits	250 m	11.58	18.05	16.92
	500 m	11.05	18.05	16.92
	1 km	10.00	18.80	18.05
	3 km	5.00	12.78	16.92
				
25 extra visits	250 m	55.26	77.44	86.84
	500 m	52.89	78.57	86.84
	1 km	47.37	78.95	86.47
	3 km	22.37	62.78	84.21
				
40 extra visits	250 m	83.95	98.50	99.25
	500 m	82.63	98.87	99.25
	1 km	78.16	98.87	99.25
	3 km	41.58	91.73	99.25

SPRING				

10 extra visits	250 m	16.50	33.93	23.93
	500 m	14.00	32.86	23.93
	1 km	12.50	32.50	25.00
	3 km	7.25	27.14	22.86
				
25 extra visits	250 m	71.25	89.29	99.29
	500 m	67.25	90.00	99.29
	1 km	58.50	88.57	99.29
	3 km	25.50	67.86	99.29
				
40 extra visits	250 m	95.50	99.64	100.00
	500 m	94.00	99.64	100.00
	1 km	92.25	99.64	100.00
	3 km	52.50	91.07	100.00

SUMMER				

10 extra visits	250 m	26.15	42.49	41.03
	500 m	25.64	43.22	41.03
	1 km	22.31	41.39	42.86
	3 km	9.74	35.90	40.66
				
25 extra visits	250 m	84.10	95.24	98.53
	500 m	83.33	95.60	98.90
	1 km	75.13	94.87	99.27
	3 km	38.97	77.66	99.27
				
40 extra visits	250 m	95.90	100.00	100.00
	500 m	95.64	100.00	100.00
	1 km	94.10	100.00	100.00
	3 km	72.05	96.34	100.00

FALL				

10 extra visits	250 m	7.95	29.30	19.05
	500 m	7.44	27.84	19.41
	1 km	6.15	28.21	19.05
	3 km	1.54	19.41	17.58
				
25 extra visits	250 m	62.56	86.81	95.97
	500 m	60.51	87.18	95.97
	1 km	51.79	84.62	96.70
	3 km	19.49	67.03	93.04
				
40 extra visits	250 m	90.51	98.63	100.00
	500 m	89.23	100.00	100.00
	1 km	87.18	100.00	100.00
	3 km	48.72	90.11	100.00

The numbers in the cells are the percentage of simulated outbreaks that exceeded the 95^th ^percentile value of M × N (M statistic × number of visits).

The simulated clusters that produced the fewest alarms were those with 10 extra visits, placed 5 km from the hospital within a circle having a radius of 3 km (sensitivity= 6%). Clusters of the same size, at the same distance, and within increasingly smaller radii also yielded few alarms. At this distance, underlying patient population density is greatest. Clusters that produced the most alarms were those with 40 extra visits, placed 50 km from the hospital, and radius size did not matter. Furthermore, sensitivity remained nearly as high (99%) when the same size clusters were placed 15 km away as long as the radius was less than 3 km. At these high rates, season had no effect on sensitivity. With a midrange cluster size (25), there was also high sensitivity (94–96%) at 50 km from the hospital with any radius size, but the effect of time of year became apparent. Winter rates (84–87%) at this distance for the four radius sizes were lower than rates for the other seasons (93–99%). Seasonal effects remained apparent as the presence of clustering became more difficult to detect. For clusters with the lowest alarm rates, those with 10 extra visits, winter sensitivity values (5–19%) were roughly half the size of summer values (10–43%). For clusters with 25 extra visits placed close to the hospital (5 km), winter rates for the different radii ranged from 22–55%, whereas summer rates ranged from 39–84%.

## Discussion

This study illustrates the importance of considering spatial information for outbreak detection, and demonstrates that using an interpoint distance distribution and precise address locations is a powerful approach. Using readily available syndromic data, quite small outbreaks would generate an alarm when cases are spatially clustered. With just 10 extra visits per week, or just over one extra visit per day, spatial clustering was detected about 20% of the time. When just under six extra clustered visits were added per day, clustering was detected about 90% of the time. Although sensitivity varies with cluster parameters, the absolute values are not of primary importance when evaluating the results because some of the simulated clusters were intended to be difficult to detect. The patterns of high and low values highlight the effects of the parameters on sensitivity.

Clusters with the lowest alarm rates were those that were small in size, large in area, and located close to the hospital where underlying patient population density was greatest. Analyses in this paper were for patients at a single hospital who tend to live close to the hospital. If data from multiple hospitals were combined, the effect of distance from the hospital might be diminished as patients are spread more uniformly over the area of coverage. Also, not all hospitals are located in dense population centers. In new locations with different population density characteristics, the sensitivity of our method will likely vary.

The extreme values of the cluster parameters were chosen specifically to test the limits of detection. We found that clusters could be too small (10 extra visits) for our method to indicate clustering, and that they could be too widely dispersed (within a circle with a 3 km radius). Midrange values in terms of size and cluster radius were sensitive to clustering in our part of the country, and may appropriately characterize the parameters of clusters expected during an actual outbreak.

The effect of season is of interest because it suggests that the choice of alarm thresholds should be tailored to time of year. However, there is some arbitrariness to seasonal boundaries based on calendar dates. For example, a week that was just prior to the date that a season changed may actually be more like the season that follows that date. Hence, the strategy for the season that follows may instead be more appropriate. Furthermore, season itself is not likely the variable of interest. Instead, variables such as changing numbers of patients or changing locations from which patients come may be the factors that affect sensitivity, rather than the season itself. And while in general these variables change over seasons, the specific time at which they change varies. For example, influenza season occurs in the winter, but does not always occur on the same dates each winter.

Ideally, a detection strategy could more precisely handle changing characteristics of the baseline data, such as the onset of influenza season. In other work, we continue to develop such strategies for the M statistic. We also continue to test its performance in areas with different geographic characteristics and with different data types such as gastrointestinal syndrome or viral tests. And finally, we are working to extend the utility of our approach by developing methods to locate where the spatial clustering occurs.

This study uses the home address of the patient, readily available in hospital information systems. Should an outbreak spread through a work or school environment, or a place of common gathering, such as a baseball stadium, the distribution of patients' home addresses may not adequately reveal the appropriate clustering. However, the methods are applicable for other patient locations [16] should more complete location information be obtained from patients in clinical settings.

## Conclusion

Measuring perturbation in the interpoint distance distribution is a sensitive method for detecting the presence of spatial clusters. When cases are clustered geographically, there is clearly power to detect clustering when the spatial distribution is represented by the M statistic, even when outbreaks are small in size. By varying independent parameters of simulated outbreaks, we have demonstrated empirically the limits of detection of different types of outbreaks.

## List of abbreviations used

ED: Emergency Department

N: number

M: M statistic

## Competing interests

The author(s) declare that they have no competing interests.

## Authors' contributions

KLO and KDM developed the study design. KLO was responsible for data acquisition and analysis, and writing the manuscript. MB and MP adapted the M statistic for biosurveillance. KDM participated in the analysis and wrote portions of the manuscript. All authors read and approved the final manuscript.

## Pre-publication history

The pre-publication history for this paper can be accessed here:


